# The effects of growth hormone on the outcomes of *in vitro* fertilization and embryo transfer in age-grouped patients with decreased ovarian reserve: a prospective cohort study

**DOI:** 10.3389/fendo.2024.1457866

**Published:** 2025-01-14

**Authors:** Jiexiu Chen, Xumei Kong, Zonghui Luan, Yu Qiu, Shiqi Chen, Jesse Li−Ling, Yan Gong

**Affiliations:** ^1^ Department of Clinical Pharmacy, Sichuan Provincial Women’s and Children’s Hospital, The Affiliated Women’s and Children’s Hospital of Chengdu Medical College, Chengdu, China; ^2^ Reproductive Medicine Center, Sichuan Provincial Women’s and Children’s Hospital, The Affiliated Women’s and Children’s Hospital of Chengdu Medical College, Chengdu, China; ^3^ School of Clinical Medicine, Chengdu Medical College, Chengdu, China; ^4^ Center of Medical Genetics, West China Second University Hospital, Sichuan University, Chengdu, China

**Keywords:** decreased ovarian reserve, *in vitro* fertilization, growth hormone, female age, live birth

## Abstract

**Background:**

Growth hormone (GH) could improve the outcomes of *in vitro* fertilization and embryo transfer (IVF-ET) in patients with decreased ovarian reserve (DOR), but which age group will benefit the most has remained controversial. This study aims to explore the outcome of IVF-ET among differently aged patients with DOR treated with GH.

**Methods:**

A total of 846 patients with DOR undergoing IVF-ET from May 2018 to June 2023 at the Reproductive Medicine Center of Sichuan Provincial Women’s and Children’s Hospital were prospectively enrolled. The patients were divided into group A (< 35 year old, *n* = 399), group B (35 ~ 40 year old, *n* = 286), and group C (> 40 year old, *n* = 161). Each group was sub-divided into the GH part and the control part, with the former receiving pretreatment with GH 4 IU/day on day 2 of the previous menstrual cycle before the injection of gonadotrophin (Gn) until the trigger day. The ovarian stimulation protocol was gonadotrophin-releasing hormone antagonist (GnRH-A) or long-acting GnRH agonist protocol. The quality of oocytes and embryos and the outcome of pregnancy were compared.

**Results:**

In group B, the number (1.16 ± 0.12 vs. 0.74 ± 0.09) and rate (34.27% vs. 23.90%) of high-quality cleavage embryos, rate of implantation (32.37% vs. 22.35%), clinical pregnancy (48.98% vs. 33.67%), and live birth (44.90% vs. 29.59%) were significantly higher, whereas the canceled oocyte retrieval rate was significantly lower (1.49% vs. 6.58%) in the GH part than those of the control part (*P* < 0.05). In group B, the duration and dose of Gn, number of oocyte retrieved, and rates of normal fertilization, cleavage embryo, blastocyst, high-quality blastocyst, and early miscarriage were not significantly different between the GH and control parts (*P* > 0.05). In groups A and C, no significant difference was detected in the quality of embryos and outcomes of embryo transfer with or without pretreatment (*P* > 0.05).

**Conclusion:**

GH could improve the quality of embryos and live birth rate for patients with DOR aged 35–40 years old.

## Introduction

The pathological feature of decreased ovarian reserve (DOR) is the reduction of ovarian follicles and female reproductive ability, with clinical characteristics as decreased serum level of anti-Müllerian hormone (AMH) and antral follicle count (AFC) (1). As reported in 2023, the overall prevalence of DOR was 37.2% among Korean women (2). About a third of patients with DOR may show poor ovarian response (POR) during *in vitro* fertilization and embryo transfer (IVF-ET), which is manifested as high risk for cycle cancellation, fewer oocytes and embryos, and lower chance for live birth (3–5). DOR has also been associated with miscarriage, recurrent pregnancy loss, and preeclampsia (6–8). The underlying reasons for DOR are complex, which may include advanced female age, ovarian and pelvic surgery, chemotherapy and radiotherapy, ovarian endometriosis cyst, smoking, pelvic infection, and environmental pollutants (9).

Some strategies have been tried to improve the outcome of IVF-ET for patients with DOR, including improved ovarian stimulation (OS) protocols, injection of luteinizing hormone (LH) during OS, pretreatment with growth hormone (GH), coenzyme Q10, and dehydroepiandrosterone (DHEA), and traditional Chinese medicine (4, 10–12). Among these, the number of studies and patients enrolled was relatively large for pretreatment with GH, a pleiotropic multifunctional hormone secreted by adenohypophyseal cells. In 1986, the first hypothesis of GH usage in IVF-ET was proposed (13). In 1991, GH co-treatment for poor responders was firstly reported (14). In 2015, GH had been recommended for poor ovarian responders by Chinese guidelines (15). Researchers have reported that GH could improve follicle development (including steroidogenesis, ovulation, and corpus luteum function), quality of oocyte (maturation and fertilization), and ovarian response to exogenous gonadotrophin (Gn) (16). Several meta-analyses have shown that GH could improve the number of oocytes retrieved and rates of fertilization, high-quality embryo, implantation and clinical pregnancy while reducing the cycle cancellation rate in patients with DOR (4, 17–20). However, whether GH can improve the live birth rate (LBR) has remained controversial. In 2020, a meta-analysis involving 1,448 poor ovarian responders undergoing IVF or intracytoplasmic injection (ICSI) has shown that GH supplementation could significantly improve the LBR [relative risk, 1.74; 95% confidence interval (CI): 1.19–2.54; *P* = 0.004] (19), while another meta-analysis involving 1,139 poor ovarian responders has found no significant difference [odds ratio, 1.34; 95% CI: 0.88–2.55; *P* = 0.17] (17). It is worth noting that both studies had not considered the age of patients.

DOR with age is a physiological phenomenon, particularly in those >35 years, and is more obvious in those >40 years. On the other hand, DOR in younger women is mainly due to pathological factors. Compared with elder women, the quality of oocytes and embryos and outcomes of IVF-ET were better in young women with DOR (21). Therefore, female age must be considered when exploring LBR among women with DOR treated with GH. The secretion of GH may decrease with aging (22); therefore, GH may be suitable for elder women, though this was not without dispute. Kevin et al. (23) retrospectively have reported that LBR was increased by GH in all age-grouped patients with a poor prognosis (<35 years, 34.8% vs. 12.3%, *P* = 0.025; 35–39 years, 24.0% vs. 2.1%, *P* = 0.001; 40–44 years, 12.5% vs. 2.4%, *P* = 0.027). Cai et al. (24) reported that LBR could be improved by GH in poor ovarian responders >35 years old (27.3% vs. 9.2%, *P* = 0.003) as well as in patients aged <35 years old (29.6% vs. 28.8%, *P* = 0.935), while Zhu et al. (25) have reported that GH could not benefit LBR among poor ovarian responders in all age groups. Therefore, we have conducted this prospective cohort study to explore the LBR among age-grouped patients with DOR treated by GH.

## Materials and methods

### Study population

From May 2018 to June 2023, patients with DOR undergoing gonadotrophin-releasing hormone antagonist (GnRH-A) or long-acting GnRH agonist (GnRH-a) protocol at the Reproductive Medicine Centre of Sichuan Provincial Women’s and Children’s Hospital were prospectively enrolled. DOR was diagnosed as AFC <7 and AMH <1.1 ng/mL (26). Patients were excluded from the study if they meet any of the following criteria: (1) uterine disease which may affect embryo implantation, such as congenital uterine malformation, submucosal or intramural uterine fibroids, intrauterine adhesion, and adenomyosis; (2) uncontrolled endocrinopathies, such as diabetes, hyperthyroidism, hypothyroidism, and/or hyperprolactinemia; (3) chromosomal aberration; (4) previous OS within 3 months; (5) fertilization with donor’s sperm or oocyte; and (6) treatment with coenzyme Q10 or DHEA and other drugs to improve the ovarian response. Any contraindication to the use of GH was excluded for patients pretreated with GH. The patients were divided into group A (<35 years old), group B (35–40 years old), and group C (>40 years old). Thereafter, each group was sub-divided into the GH part and the control part. The AFC, body mass index (BMI), and serum levels of follicular stimulation hormone (FSH), LH, estradiol (E_2_), progesterone (P), total testosterone (TT), prolactin (PRL), and AMH were measured as described elsewhere (27).

### Ovarian stimulation

With the GnRH-A protocol, the patients had received a daily injection of 225–300 IU recombinant follicular stimulation hormone (rFSH, Gonal-F, Merck-Serono KGaA., Darmstadt, Germany; Jinsai Heng, Jinsai Pharmaceuticals, China; Puregon^®^, Merck Sharp & Dohme, USA) from day 2 to 3 of the menstrual cycle until the trigger day. The dose of rFSH was adjusted according to the follicular development and serum level of E_2_. When the diameter of the dominant follicle had reached 14 mm or when the serum level of LH was ≥10 mIU/mL, 0.25 mg GnRH-A (Ganirelix, Ocalon, USA) was injected subcutaneously daily until the trigger day. With the GnRH-a protocol, 3.75 mg leuprorelin acetate (Shanghai Livzon Pharmaceutical, China) was injected once on days 2 to 3 of the menstrual cycle. At 28–35 days later, when the pituitary was downregulated (serum FSH and LH <5 mIU/mL, E_2_ <50 pg/mL, and diameter of follicle <5 mm), 225–300 IU of rFSH was injected daily until the trigger day.

With both OS protocols, when the diameter of at least one or two follicles had reached 18 mm, 250 μg of recombinant human chorionic gonadotrophin (rHCG, Merck-Sheranova, Germany) was injected, and the oocytes were retrieved under transvaginal ultrasound guidance after 36.5 h. With the GnRH-A protocol, some patients were injected with a dual trigger, i.e., 0.1 mg triptorelin (Changchun GeneScience Pharmaceuticals Co., Ltd., Changchun, Jilin, China) in combination with 4,000 IU HCG (Shanghai Livzon Pharmaceutical, China). The patients from the GH part were subcutaneously injected with 4 IU/day recombinant GH (rGH, Changchun GeneScience Pharmaceuticals Co., Ltd., Changchun, Jilin, China) on day 2 of the previous menstrual cycle before rFSH until the trigger day, and those from the control part had received no pretreatment. Ovarian hyper-stimulation syndrome (OHSS) was diagnosed and graded according to Navot et al. (28). The reasons for the cancellation of oocyte retrieval had included follicular growth failure (10 days after OS, leading to follicle diameter <10 mm) and premature ovulation before oocyte retrieval.

### *In vitro* fertilization and embryo culture

Following oocyte retrieval, IVF or ICSI was conducted according to the quality of sperm. Mature oocyte was defined as being at the metaphase II (MII) stage with the first polar body visible in the cytoplasm. Normal fertilized oocyte was defined as containing two pronuclei (2PN). Embryos after fertilization were cultured in sequential G1-plus/G2-plus medium (Vitrolife, Sweden) at 37°C under 6% CO_2_ and 5% O_2_. Day 3 cleavage embryo was scored based on the number of blastomeres and degree of fragmentation, and the high-quality embryo was categorized as grade A/B (29). On day 5 or 6, the blastocyst was scored by Gardner and Schoolcraft’s system, and 4BB or better was considered as a high-quality blastocyst (29).

### Fresh embryo transfer and luteal phase support

All embryos were frozen on any of the following conditions: no transplantable embryo formation, abnormal endometrium (thickness <7 mm or with abnormal ultrasonic image), serum P >1.5 ng/mL on the trigger day, and abandonment of fresh ET of the patients. All of the remaining patients had undergone fresh embryo transfer with one or two day 3 cleavage embryos with the highest morphological grade. The remaining embryos were frozen or cultured to the blastocyst stage to be frozen based on the choice of patients. After oocyte retrieval, vaginal progesterone gel (90 mg) (Crinone, Merck, Germany) was given daily, accompanied with dydrogesterone (30 mg/day) (Abbott, The Netherlands) orally from the ET day until the 12th gestational week as luteal phase support. Serum hCG was measured 12 days after ET, and hCG positivity was considered when hCG >5 IU/mL. Clinical pregnancy was defined as the identification of a gestational sac with an embryo showing cardiac activity. Early miscarriage was defined as the loss of pregnancy before the 12th gestational week. Live birth was defined as the delivery of a live fetus at ≥28 weeks of gestation.

### Outcomes

The primary outcome was LBR per fresh ET cycle. The secondary outcomes were the rates of MII, 2PN, cleavage embryo, high-quality cleavage embryo, blastocyst, high-quality blastocyst, implantation, clinical pregnancy, early miscarriage, and canceled oocyte retrieval cycle. MII rate was calculated as the number of MII oocytes divided by the number of oocytes retrieved, whereas 2PN rate was calculated as the number of 2PN divided by the number of MII oocytes. The rates of cleavage embryo and high-quality cleavage embryo were calculated as the number of day 3 cleavage embryos or high-quality cleavage embryos divided by the number of cleaved embryos on day 2, respectively. The rates of blastocyst and high-quality blastocyst were calculated as the numbers of usable blastocysts or high-quality blastocysts divided by the number of day 3 cleavage embryos for blastocyst culture, respectively. Implantation rate (IR) was calculated as the number of gestational sacs divided by the number of transferred embryos. CPR and LBR were calculated as the numbers of clinical pregnancy cycles or live birth divided by the number of embryo transfer cycles. Multiple pregnancy rate was calculated as the number of multiple pregnancy cycles divided by the clinical pregnancy cycles.

### Statistical analysis

Statistical analysis was performed by using SPSS version 26.0 software (IBM, Armonk, NY, USA). Continuous variables were expressed as means with standard error and compared by using Student’s *t*-test or Mann–Whitney’s *U*-test for normal distribution or abnormal distribution data, respectively. Categorical variables were presented as numbers with percentages and compared by using chi-square or Fisher’s exact tests. A two-tailed *P <*0.05 was regarded as statistically significant. No *a priori* sample size calculation was performed because we decided to include all cases that had met the inclusion criteria in the specified time period.

## Results

### Basal characteristics of the study population

**Figure 1:**
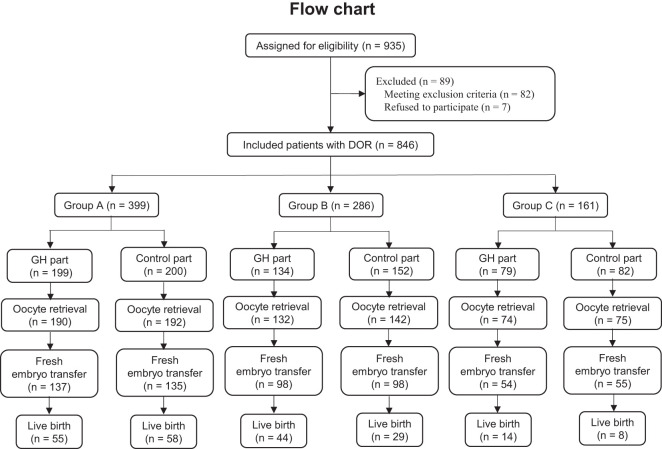
Flowchart of the recruitment process.

A total of 846 patients were enrolled and divided into group A (GH part, *n* = 199; control part, *n* = 200), group B (GH part, *n* = 134; control part, *n* = 152), and group C (GH part, *n* = 79; control part, *n* = 82), while 89 patients were excluded. A flowchart of the recruitment process is shown in **Figure 1**. For each group, the age, duration and type of infertility, BMI, AFC, AMH, and serum level of basal sex hormone were not significantly different between the GH and control parts (*P* > 0.05) (**Table 1**).

**Table 1:** Basal characteristics of the study populations.

	Group A				Group B				Group C			
	GH (*n* = 199)	Control (*n* = 200)	*T*/*χ* ^2^	*P*	GH (*n* = 134)	Control (*n* = 152)	*T*/*χ* ^2^	*P*	GH (*n* = 79)	Control (*n* = 82)	*T*/*χ* ^2^	*P*
Age (years)	30.84 (0.19)	30.46 (0.21)	1.361	0.174	37.34 (0.15)	37.10 (0.14)	1.208	0.228	42.43 (0.19)	42.83 (0.21)	-1.398	0.164
Duration of infertility (years)	3.48 (0.20)	3.15 (0.18	1.219	0.224	4.05 (0.34)	4.95 (0.36)	-1.817	0.070	4.29 (0.52)	4.30 (0.51)	-0.016	0.987
Type of infertility			2.138	0.144			0.505	0.477			0.102	0.750
Primary	54.27% (108/199)	61.50% (123/200)			32.84% (44/134)	28.95% (44/152)			16.46% (13/79)	14.63% (12/82)		
Secondary	45.73% (91/199)	38.505 (77/200)			67.16% (90/134)	71.05% (108/152)			83.54% (66/79)	85.37% (70/82)		
BMI (kg/m^2^)	22.06 (0.20)	22.51 (0.22)	-1.514	0.131	22.96 (0.29)	22.34 (0.21)	1.729	0.085	23.02 (0.28)	22.61 (0.29)	1.022	0.308
AFC (*n*)	5.90 (0.16)	6.23 (0.18)	-1.343	0.180	6.26 (0.21)	5.99 (0.17)	1.032	0.303	4.91 (0.23)	4.60 (0.21)	1.023	0.308
AMH (ng/mL)	0.76 (0.03)	0.79 (0.03)	-0.757	0.450	0.81 (0.04)	0.77 (0.03)	0.763	0.446	0.65 (0.05)	0.65 (0.05)	-0.005	0.996
Basal FSH (IU/mL)	9.50 (0.33)	8.78 (0.25)	6.535	0.085	8.93 (0.37)	8.73 (0.30)	0.438	0.662	10.19 (0.58)	9.29 (0.41)	1.270	0.206
Basal LH (IU/mL)	4.56 (0.17)	4.61 (0.17)	-0.197	0.844	5.01 (0.25)	4.64 (0.17)	1.249	0.213	5.32 (0.29)	5.08 (0.22)	0.675	0.500
Basal E_2_ (pg/mL)	44.57 (2.30)	46.04 (2.77)	-0.406	0.685	49.90 (3.91)	43.73 (2.24)	1.409	0.160	49.61 (2.90)	51.06 (3.03)	-0.345	0.731
Basal P (ng/mL)	0.46 (0.02)	0.44 (0.02)	1.058	0.291	0.45 (0.02)	0.43 (0.02)	0.895	0.372	0.45 (0.04)	0.39 (0.03)	1.204	0.230
TT (ng/mL)	0.21 (0.01)	0.22 (0.01)	-0.919	0.359	0.20 (0.02)	0.20 (0.01)	0.036	0.971	0.22 (0.02)	0.22 (0.02)	-0.078	0.938
PRL (μIU/mL)	336.94 (14.05)	322.09 (13.62)	0.758	0.450	319.12 (20.15)	317.10 (15.10)	0.082	0.935	324.26 (32.24)	302.06 (23.07)	0.555	0.581

The continuous variables were described by using mean (standard error of mean).

### Ovarian stimulation

For group B, the oocyte retrieval canceled rate was significantly lower for the GH part compared with the control part (*P* < 0.05), whereas the proportion of OS protocol, dose and duration of rFSH, serum levels of E_2_, LH, and P on the trigger day, type of trigger, and fertilization were not significantly different (*P* > 0.05). For groups A and C, the above mentioned characteristics were not significantly different between the GH and the control parts (*P* > 0.05) (**Table 2**). No moderate or severe OHSS was noted in all patients.

**Table 2:** Outcome of ovarian stimulation.

	Group A				Group B				Group C			
	GH (*n* = 199)	Control (*n* = 200)	*T*/*χ* ^2^	*P*	GH (*n* = 134)	Control (*n* = 152)	*T*/*χ* ^2^	*P*	GH (*n* = 79)	Control (*n* = 82)	*T*/*χ* ^2^	*P*
COH protocol			1.108	0.292			1.265	0.261			2.939	0.086
GnRH antagonist	53.27% (106/199)	58.50% (117/200)			65.67% (88/134)	59.21% (90/152)			93.67% (74/79)	85.37% (70/82)		
GnRH agonist	46.73% (93/199)	41.50% (83/200)			34.33% (46/134)	40.79% (62/152)			6.33% (5/79)	14.63% (12/82)		
rFSH dose (IU)	2,738.57 (65.89)	2,729.81 (74.96)	0.088	0.930	2,662.22 (92.26)	2,815.93 (86.91)	-1.213	0.226	2,368.83 (100.30)	2,530.49 (109.24)	-1.088	0.278
rFSH duration (day)	10.22 (0.18)	9.78 (0.18)	1.735	0.083	9.64 (0.24)	10.03 (0.22)	-1.197	0.232	8.62 (0.26)	9.11 (0.32)	-1.176	0.241
Serum E_2_ on HCG day (pg/mL)	1,355.31 (72.60)	1,256.39 (56.85)	1.076	0.283	1,207.72 (57.03)	1,284.89 (70.18)	-0.843	0.400	938.81 (53.16)	1,035.34 (106.75)	-0.809	0.420
Serum LH on HCG day (IU/mL)	3.08 (0.27)	3.85 (0.33)	-1.813	0.071	3.87 (0.29)	3.41 (0.37)	0.970	0.333	5.60 (0.50)	4.67 (0.49)	1.327	0.186
Serum P on HCG day (ng/mL)	0.73 (0.03)	0.80 (0.03)	-1.677	0.094	0.77 (0.04)	0.75 (0.04)	0.443	0.658	0.69 (0.06)	0.57 (0.04)	1.626	0.106
Type of trigger			0.491	0.483			1.715	0.190			1.986	0.159
HCG	90.00% (171/190)	87.76% (172/196)			89.55% (120/134)	84.25% (123/146)			84.21% (64/76)	75.00% (57/76)		
Dual trigger	10.00% (19/190)	12.24% (24/196)			10.45% (14/134)	15.75% (23/146)			15.79% (12/76)	25.00% (19/76)		
Protocol of fertilization			0.858	0.354			0.458	0.498			0.972	0.324
IVF	82.26% (153/186)	85.79% (157/183)			77.95% (99/127)	81.29% (113/139)			84.93% (62/73)	78.57% (55/70)		
ICSI	17.74% (33/186)	14.21% (26/183)			22.05% (28/127)	18.71% (26/139)			15.07% (11/73)	21.43% (15/70)		
Oocyte retrieval cancellation rate (%)	4.52% (9/199)	4.00% (8/200)	0.670	0.796	1.49% (2/134)	6.58% (10/152)	4.584	0.032	6.33% (5/79)	8.54% (7/82)	0.284	0.594

The continuous variables were described by using mean (standard error of mean).

For group A, nine patients from the GH part had canceled oocyte retrieval (4 for follicular growth failure and 5 for premature ovulation), compared with 8 from the Control part (4 for follicular growth failure and 4 for premature ovulation). For group B, 2 patients from the GH part had canceled oocyte retrieval (1 for follicular growth failure and 1 for premature ovulation), compared with 10 from the Control part (5 for follicular growth failure and 5 for premature ovulation). For group C, 5 patients from the GH part had canceled oocyte retrieval (2 for follicular growth failure and 3 for premature ovulation), compared with 7 from the Control part (5 for follicular growth failure and 2 for premature ovulation).

### Outcomes of oocytes and embryos

A total of 805 patients had undergone oocyte retrieval. For group B, the number and rate of high-quality cleavage embryo was significantly greater in the GH part compared with the Control part (*P* < 0.05), whereas the number of oocytes retrieved, numbers and rates of MII, 2PN, cleavage embryos, blastocysts and high-quality blastocysts were not significantly different (*P* > 0.05). For groups A and C, above characteristics were not significantly different between the GH and the Control parts (*P* > 0.05) (**Table 3**).

**Table 3:** Outcomes of oocytes and embryos.

	Group A				Group B				Group C			
	GH (*n* = 190)	Control (*n* = 192)	*T*/*χ* ^2^	*P*	GH (*n* = 132)	Control (*n* = 142)	*T*/*χ* ^2^	*P*	GH (*n* = 74)	Control (*n* = 75)	*T*/*χ* ^2^	*P*
Oocytes retrieved (*n*)	4.79 (0.20)	4.79 (0.20)	0.011	0.992	4.76 (0.27)	4.58 (0.22)	0.525	0.600	3.35 (0.31)	3.03 (0.29)	0.776	0.439
MII oocyte (*n*)	4.10 (0.18)	4.27 (0.19)	-0.639	0.523	4.18 (0.22)	3.92 (0.20)	0.882	0.379	2.76 (0.24)	2.72 (0.21)	0.111	0.912
2PN (*n*)	2.85 (0.15)	2.80 (0.14)	0.247	0.850	2.84 (0.19)	2.70 (0.16)	0.594	0.553	2.08 (0.21)	1.91 (0.21)	0.571	0.569
Cleavage embryo (*n*)	2.84 (0.16)	2.91 (0.15)	-0.318	0.751	2.98 (0.18)	2.66 (0.16)	1.360	0.175	1.97 (0.19)	2.00 (0.21)	-0.099	0.921
High-quality cleavage embryo (*n*)	1.03 (0.10)	1.12 (0.09)	-0.642	0.521	1.16 (0.12)	0.74 (0.09)	2.815	0.005	0.69 (0.09)	0.52 (0.09)	1.364	0.175
Usable blastocyst (*n*)	0.38 (0.07)	0.47 (0.08)	-0.858	0.392	0.43 (0.10)	0.28 (0.07)	1.189	0.235	0.24 (0.11)	0.04 (0.03)	1.843	0.068
High-quality blastocyst (*n*)	0.12 (0.04)	0.09 (0.03)	0.660	0.510	0.15 (0.04)	0.11 (0.04)	0.703	0.482	0.09 (0.05)	0	1.736	0.086
MII rate (%)	83.75% (763/911)	84.89% (781/920)	0.448	0.503	84.55% (531/628)	83.855 (545/650)	0.120	0.729	80.24% (199/248)	82.82% (188/227)	0.522	0.470
2PN rate (%)	58.38 (530/908)	56.02% (512/914)	1.030	0.310	58.23% (361/620)	58.05% (375/646)	0.004	0.949	59.43% (145/244)	61.11% (132/216)	0.136	0.713
Cleavage embryo rate (%)	87.13% (528/606)	87.21% (532/610)	0.002	0.965	88.34% (379/429)	85.85% (370/431)	1.194	0.275	88.20% (142/161)	90.79% (138/152)	0.556	0.456
High-quality cleavage embryo rate (%)	31.68% (192/606)	33.61 (205/610)	0.511	0.475	34.27% (147/429)	23.90% (103/431)	11.208	0.001	31.06% (50/161)	23.68% (36/152)	2.132	0.144
Blastocyst rate (%)	43.75% (70/160)	51.51 (86/167)	1.966	0.161	49.54% (54/109)	41.05% (39/95)	1.475	0.225	39.29% (11/28)	15.38% (2/13)	2.342	0.126
High-quality blastocyst rate (%)	14.38% (23/160)	10.18% (17/167)	1.340	0.247	17.43% (19/109)	15.79% (15/95)	0.099	0.754	14.29% (4/28)	0/13	2.058	0.151

The continuous variables were described by using mean (standard error of mean).

### Outcomes of fresh embryo transfer

For group A, 53 patients had canceled fresh embryo transfer (ET) in the GH part (27 for without transplantable embryo, 13 for abnormal endometrium, four for elevated P level, and nine for patient’s abandonment) and 57 patients in the control part (27 for without transplantable embryo, 22 for abnormal endometrium, three for elevated P level, and 5 for patient’s abandonment). For group B, 34 patients had canceled fresh ET in the GH part (15 for without transplantable embryo, 15 for abnormal endometrium, two for elevated P level, and two for patient’s abandonment) and 44 patients in the control part (19 for without transplantable embryo, 15 for abnormal endometrium, one for elevated P level, and nine for patient’s abandonment). For group C, 20 patients had canceled fresh ET in the GH part (14 for without transplantable embryo and six for abnormal endometrium), along with 20 patients in the control part (14 for without transplantable embryo, four for abnormal endometrium, and two for patient’s abandonment).

A total of 554 patients had undergone fresh ET. In group B, the rates of implantation, clinical pregnancy, and live birth were significantly higher in the GH part than the control part (*P* < 0.05), whereas the endometrial thickness, number of embryos transferred, proportion of at least one high-quality embryo transfer cycle, multiple pregnancy and early miscarriage, gestational age at delivery, and birth weight and height of the infants were not significantly different (*P* > 0.05). For groups A and C, the abovementioned characteristics were not significantly different between the GH and the control parts (*P* > 0.05) (**Table 4**).

**Table 4:** Outcomes of fresh embryo transfer.

	Group A				Group B				Group C			
	GH (*n* = 137)	Control (*n* = 135)	*T*/*χ* ^2^	*P*	GH (*n* = 98)	Control (*n* = 98)	*T*/*χ* ^2^	*P*	GH (*n* = 54)	Control (*n* = 55)	*T*/*χ* ^2^	*P*
Endometrial thickness (mm)	10.93 (0.19)	10.75 (0.21)	0.645	0.520	10.22 (0.24)	10.55 (0.27)	-0.885	0.377	9.32 (0.29)	9.48 (0.30)	-0.386	0.700
Number of embryo transferred (*n*)	1.80 (0.04)	1.78 (0.04)	0.358	0.721	1.77 (0.04)	1.83 (0.04)	-1.061	0.290	1.72 (0.06)	1.56 (0.07)	1.462	0.159
At least one high-quality embryo transfer cycle (%)	52.55% (72/137)	60.77% (82/135)	1.855	0.173	53.06% (52/98)	51.02% (50/98)	0.082	0.775	37.04% (20/54)	41.82% (23/55)	0.261	0.610
Implantation rate (%)	32.11% (79/246)	36.67% (88/240)	1.116	0.291	32.37% (56/173)	22.35% (40/179)	4.456	0.035	21.51% (20/93)	15.12% (13/86)	1.213	0.271
Clinical pregnancy rate (%)	45.26% (62/137)	48.89% (66/135)	0.360	0.584	48.98% (48/98)	33.67% (33/98)	4.734	0.030	31.48% (17/54)	20.00% (11/55)	1.882	0.170
Multiple pregnancy rate (%)	27.42% (17/62)	33.33% (22/66)	0.528	0.468	16.67% (8/48)	21.21% (7/33)	0.268	0.605	17.65% (3/17)	18.18% (2/11)	0.001	0.971
Early miscarriage rate (%)	4.84% (3/62)	9.09% (6/66)	0.884	0.347	8.33% (4/48)	6.06% (2/33)	0.147	0.701	17.65% (3/17)	27.27% (3/11)	0.368	0.544
Live birth rate (%)	40.15% (55/137)	42.96% (58/135)	0.222	0.637	44.90% (44/98)	29.59% (29/98)	4.911	0.027	25.93% (14/54)	14.55% (8/55)	2.191	0.139
Gestational age at delivery (week)	37.71 (0.33)	37.88 (0.31)	-0.374	0.709	38.07 (0.24)	37.97 (0.36)	0.246	0.806	38.64 (0.31)	37.88 (0.44)	1.462	0.159
Birth weight (gram)	2,802.59 (84.90)	2,810.74 (79.24)	-0.070	0.944	2,965.16 (71.16)	3,116.94 (90.29)	-1.326	0.188	3,048.82 (110.99)	2,879.00 (94.22)	1.046	0.306
Birth height (cm)	47.96 (0.41)	47.89 (0.37)	0.118	0.906	48.51 (0.49)	48.70 (0.47)	-0.262	0.794	49.24 (0.40)	49.10 (0.43)	0.219	0.828

The continuous variables were described by using mean (standard error of mean).

For group A GH part, two had ectopic pregnancies, one and two pairs of twins had undergone early and late miscarriage, respectively. In the group A control part, one pair of twins had undergone early miscarriage, one pair of twins and one single pregnancy had undergone late miscarriage, and three pairs of twins had undergone single fetal demise. For group B GH part, one pair of twins had undergone early miscarriage. For the GH B control part, one had undergone ectopic pregnancy, one pair of twins had undergone late miscarriage, and two pairs of twins had undergone single fetal demise. The mean gestational age at delivery was 38 weeks (28–42 weeks). The mean birth weight was 2,898 g (1,300–4,300 g), and the birth height was 48 cm (27–59 cm). None of the 255 live births (131 boys and 124 girls) was found with a birth defect.

## Discussion

In this study, we have found that the number and rate of high-quality cleavage embryo, IR, CPR, and LBR were increased, while the proportion of oocyte retrieval canceled cycle was decreased by GH among patients with DOR aged 35–40 years, whereas there was no significant difference in those under 35 or over 40.

GH plays a vital role in follicle development, including steroidogenesis, oocyte maturation, ovulation, and fertilization (16). The secretion of GH and expression of FSH and LH receptors in granulosa cells (GCs) will decrease along with age (30). Exogenous GH could enhance the responsiveness and sensitivity of GCs to Gn by upregulating the expression of FSH and LH receptors in older women with DOR (31). This may explain why the cycle cancellation rate was decreased by GH in patients with DOR aged 35–40, while no difference was found among those aged <35 years old, which is in keeping with reports by others (25, 30). For patients with DOR aged >40 years old, the cycle cancellation rate was not decreased by GH. This may be attributed to the change of GH receptors (GHR) along with aging. Regan et al. (32) have reported an increased expression of GHR along with aging in patients with normal ovarian reserve, while it was decreased in patients with DOR aged 39–45. Therefore, for patients with DOR aged >40 years, after the GHR was occupied by endogenous GH, the excessive exogenous GH could hardly produce an effect. Nevertheless, Lan et al. (33) have found that the cycle cancellation rate could be decreased by GH in patients with DOR >40 years old. The inconsistency may be attributed to their younger age (41 years old vs. 42 years old), higher dose of GH (8 IU/day vs. 4 IU/day), and larger sample size (432 patients vs. 161 patients) of their patients compared with ours.

The quality of oocytes was decreased with aging, manifested as abnormality of morphology, chromosome and meiosis, decreased function of mitochondrial, failure of fertilization, cleavage, and embryo implantation, and increased aneuploid embryos (34). Studies have reported that GH could improve the quality of GCs and oocytes in older patients via GHR and insulin-like growth factor-I (IGF-I) and improve the steroidogenesis of GCs, mitochondrial function of GCs and oocytes, and development potential of oocytes (35–37). Therefore, we have found that the number and rate of high-quality cleavage embryo were increased by GH in patients with DOR aged 35–40 years old, but not in those <35 years old, which is in keeping with reports by others (25, 38). We have also found that the rates of high-quality cleavage embryo, blastocyst, and high-quality blastocyst were increased by GH, but without significant difference in those with DOR >40 years old, which is consistent with those reported by Guo et al. (39). The lack of benefit of GH for patients >40 years old may be attributed to the fact that, for patients >40 years, the quality of oocytes and embryos may decrease sharply (34) and could not be significantly recovered by GH alone. Moreover, the effect of GH was weak due to the decreased expression of GHR in patients aged 39–45 years old (33). Furthermore, the lack of significance may also be attributed, in part, to the small sample size of such patients in our study. It is worth noting that Kevin et al. (23) have reported that patients >40 years old had benefited from GH. The inconsistency may be attributed to the younger age of the patients in their study (39 years old vs. 42 years old).

In keeping with the improved quality of embryos, we found that the IR, CPR, and LBR were increased by GH in patients with DOR aged 35–40 years old, but not in those aged <35 years old. Dogan et al. (40) also noted that GH may increase the LBR in patients with DOR aged 34–39 years. Norman et al. (41) have conducted a randomized clinical trial on poor ovarian responders aged 35–40 years old and found that LBR was not improved by GH. Liu et al. (42) reported that GH could improve LBR among poor responders both <35 and ≥35 years old. The effect of GH on LBR was also controversial in patients >40 years old. Kevin et al. (23) have reported that GH increased LBR (12.5% vs. 2.4%) for poor ovarian responders >40 years old. Feng et al. (43) have reported that GH was an independent factor of LBR in women aged 35–43 years old. Meanwhile, we found that LBR was increased by GH in patients with DOR >40 years old, but without significance in this study. The controversy may be attributed to the small sample size in our study, particularly for patients >40 years old. The effect on outcomes of IVF-ET was associated with the dose and duration of GH (44), while the dose (1–12 IU/day) and duration (10 days–6 weeks) of GH were different in these studies. The patients were different in these reports (DOR, POR diagnosed with Bologna criteria, or poor prognosis according to the POSEIDON criteria). Despite the controversy, the patients with DOR aged 35–40 years old have been the biggest beneficiary of GH. Keane et al. (23) also reported that LBR was increased by 3.81, 14.68, and 5.79 times in poor ovarian responders aged <35, 35–39, and 40–44 years old.

In summary, we have found that GH was beneficial for patients with DOR older than 35 years, especially those 35–40 years old, though this result should be interpreted with caution considering the small sample size (especially for those >40 years), this being a single-centered study, and the inherent limitation of the observational study.

## Data Availability

The original contributions presented in the study are included in the article/supplementary material. Further inquiries can be directed to the corresponding author.
